# Effects of Multidimensional Treatment Foster Care for Preschoolers (MTFC-P) for Young Foster Children with Severe Behavioral Disturbances

**DOI:** 10.1007/s10826-017-0661-4

**Published:** 2017-02-14

**Authors:** Caroline S. Jonkman, Carlo Schuengel, Mirjam Oosterman, Robert Lindeboom, Frits Boer, Ramon J. L. Lindauer

**Affiliations:** 1 Department of Child and Adolescent Psychiatry, Academic Medical Center, University of Amsterdam, Amsterdam, The Netherlands; 2 De Bascule, Academic Center for Child and Adolescent Psychiatry, Amsterdam, The Netherlands; 3 Department of Clinical Child and Family Studies and the EMGO Institute for Health and Care Research, VU University, Amsterdam, The Netherlands; 4 Department of Clinical Methods and Public Health, Academic Medical Center, University of Amsterdam, Amsterdam, The Netherlands

**Keywords:** Behavioral problems, Attachment, Trauma, Foster care, Randomized controlled trial

## Abstract

Multidimensional Treatment Foster Care for Preschoolers (MTFC-P) has thus far only been tested for diminishing behavior problems in the US. This study tested relative efficacy of MTFC-P on multiple outcomes against treatment as usual in the Netherlands (TAU; Study I), and regular foster care (Study II). The sample included 55 children that received MTFC-P, 23 children received TAU and 30 children from regular foster care (RFC). Changes in behavioral and relationship functioning, trauma symptoms, hypothalamic-adrenal-pituitary (HPA-) axis functioning, and caregiving stress were assessed via questionnaires, interviews, and salivary cortisol. Outcomes of Study I were evaluated using a randomized controlled design and quasi-experimental design, outcomes of Study II according to non-equivalent group comparison. No evidence was found for relative efficacy of MTFC-P over TAU. A treatment effect was found on trauma symptoms, in favor of TAU. Outcomes of Study II revealed that whereas caregiving stress and secure base distortions were significantly more severe at baseline in MTFC-P compared to RFC, post treatment differences were no longer significant. However, percentages of symptoms of disinhibited attachment and attachment disorder were nearly equal between groups at baseline, while post treatment percentages indicated significantly more symptoms in MTFC. In addition, results revealed a significant difference in the severity of externalizing problems post treatment, in favor of RFC. The results obtained within this study indicate that children in MTFC-P and usual treatment foster care in the Dutch context improved similarly, thus not showing the same advantages that MTFC-P has demonstrated in the US. Results should be interpreted with caution due to lower than planned power. Findings underscore the challenges of testing novel treatments across contexts with highly different child welfare provisions.

## Introduction

Almost three percent of all children in the Netherlands in 2010 have been exposed to one or multiple forms child abuse and neglect (Euser et al. 2013). Currently, over 20,000 children stay in the Dutch foster care system (Foster Care Netherlands 2015) and like in many other countries this number is on the rise (Fernandez and Barth 2010). Placement in foster care usual follows from a court-mandated out-of-home placement due to child abuse and neglect (Strijker and Knorth 2009). Also in other countries children in foster care usually have a history of physical abuse, sexual abuse, or neglect (Minnis et al. 2006). Placement in foster care is aimed to protect children from further harm. However, disruption of relationships with birth parents adds to the adverse experiences that may have occurred. As a consequence, behavioral functioning of children in foster care often becomes problematic, while many suffer from posttraumatic stress symptoms (Kolko et al. 2010), disorganized attachment (Van den Dries et al. 2009), and high frequencies of clinical symptoms of attachment disorder (Bruce et al. 2009; Gleason et al. 2011; Zeanah et al. 2005). In addition to deviant socio-emotional development, studies have documented abnormalities in neurobiological functioning in children in foster care as well (Bruce et al. 2009; Dozier et al. 2006; Gunnar et al. 2001; Gunnar and Vazquez 2001). Problematic behavioral functioning negatively affects parenting of foster parents (Vanderfaeillie et al. 2012), hampers the formation of secure attachment relationships (Dozier and Rutter 2008), and may cause placement failure (Oosterman et al. 2007b). Therefore, the need is high for effective treatment options for children in foster care to address emerging behavior problems.

Theories of social learning and coercive family processes have successfully explained behavioral problems in young children and guided effective interventions (Brestan and Eyberg 1998; DeGarmo et al. 2004; Patterson 1982; Patterson et al. 1992). According to these theories, problematic behavior is modeled and reinforced by escalating coercive parent-child interactions. The Oregon Social Learning Center developed Multidimensional Treatment Foster Care for Preschoolers (MTFC-P; Fisher et al. 1999). Within this treatment model parent training for foster parents is combined with effective behavioral interventions for young children (aged 3–7 years). Children are placed with well-trained and extensively supported foster parents. These foster parents are taught to ignore inappropriate behaviors, abandon coercive strategies, and if needed respond with non-harsh discipline strategies (for example, time-outs). MTFC-P includes social skills training for children, with an extensive focus on positive feedback in response to socially normative behavior. The effectiveness of the social learning strategies used within MTFC-P has been documented in prior research (Brestan and Eyberg 1998; DeGarmo et al. 2004). However, it was not certain that effectiveness would sustain when strategies are applied to families in foster care.

In a preliminary study, Fisher and Kim (2007) reported improved behavioral functioning of foster children in MTFC-P. Results were repeated in a subsequent randomized controlled trial, which found a decrease in resistant behavior. This time-effect was however found in both MTFC-P and regular foster care conditions. The first treatment-effects demonstrated in a randomized controlled trial were published in 2005, using data of 90 children (Fisher et al. 2005). Children assigned to the MTFC-P intervention (*n* = 47) experienced less placement failures than children in regular foster care (*n* = 43). Besides the effects on behavior and placement stability, effectiveness has been shown on secondary outcomes based on data from a randomized control trial, including 117 children in foster care (57 in MTFC-P, 60 in regular foster care). The results published in 2007 suggested that MTFC-P was superior above regular foster care in reversing the abnormalities in HPA-axis after adverse early life experiences (Fisher et al. 2007), and improving attachment related behavior (Fisher and Kim 2007). Success of MTFC-P was ascribed to the supportive caregiving environment brought about by the intervention. A later report found immediate decrease in caregiving stress in MTFC-P, not in regular foster care. This report also provided evidence that simultaneously with decreases in caregiving stress, children were protected against maladaptive HPA-axis functioning associated with caregiving stress (Fisher and Stoolmiller 2008). The question arises whether MTFC-P strategies are equally effective when applied in foster care agencies outside the US.

With the exception of a pilot investigation in the Netherlands (Jonkman et al. 2012), effect studies have thus far been reported only for children in US foster care. Given that relative efficacy of manualized youth psychosocial treatment protocols developed in the US in general has been found to be weaker when studies on those same protocols were conducted outside the US (Weisz et al. 2013), implementation of MTFC-P in others countries should therefore be carefully evaluated. For example, in Sweden and England randomized controlled trials have been performed that examined the effectiveness of the adolescent version of MTFC. In 2011 results of the Swedish trial showed a positive effect on externalizing behaviors, but effects were not as strong as in the US trials (Westermark et al. 2011). Researchers in England did not find evidence that MTFC was superior to treatment foster care as usual (Green et al. 2014). However, results were inconclusive based on the low statistical power and imbalances between the two groups at baseline. The lack of high-quality research enables us to make a statement about the sustainability of effectiveness when MTFC-P is applied in foster care other countries outside the US. Research on MTFC-P in other contexts is necessary given the important differences between countries in the organization and quality of foster care and adjunctive support (Strijker et al. 2008). Clinical care in the Netherlands consists of Therapeutic Foster Care intervention. Rather than focusing primarily on behavior problems and indirectly achieving secondary outcomes as MTFC-P, usual treatment foster care may tailor its focus on trauma, attachment, and caregiving if these are perceived as the most prominent needs of the individual child and/or the foster family.

This research is divided into two separate studies. In Study I, the relative efficacy of MTFC-P was evaluated as a treatment option for children in foster care with severe behavioral problems, as compared to usual treatment foster care (Therapeutic Foster Care intervention). The hypothesis was that MTFC-P was more effective in terms of behavioral improvement due to the strong focus on effective behavioral strategies. Secondary outcomes such as symptoms of attachment disorder, trauma symptoms, caregiving stress, and HPA-axis functioning of children in foster care and their foster parents were included as well. However, for secondary outcomes the study was more exploratory, given that these outcomes may only be indirectly affected by MTFC-P. In Study II, the outcomes of MTFC-P were compared to outcomes of children placed in regular foster care. In contrast to children in treatment foster care, children in regular foster care receive a minimum of care, which is more similar to the comparison group used in the US MTFC-P studies. However, given the availability of treatment foster care in the Netherlands, the population in regular foster care was also expected to be less problematic than the population referred for MTFC-P. The hypothesis was similar to studies in the US where MTFC-P was compared to regular foster care. We expected that children in the MTFC-P condition would show a larger decline in problematic outcomes than children in regular foster care, taking into account factors that may have determined referral for a treatment program rather than regular foster care. The importance of this latter question not only regards the comparison with US studies on MTFC-P efficacy, but also the evaluation of the treatment goals of MTFC-P, which includes reduction of symptoms that allow end of treatment and placement in regular foster care.

## Study 1

Examination of effectiveness was planned as a Two-Group Comparison Repeated Measures Design with random assignment (Randomized Controlled Trial; RCT, Trial Registration: NTR1747). Children were randomly assigned to MTFC-P or treatment foster care as usual (TAU) in a 1:1 ratio, based on the principles of Zelen’s design of pre-randomization wherein randomization is conducted prior to informed consent (Zelen 1979). The full trial protocol was registered and published (Jonkman et al. 2013) and together with important deviations from the original protocol, approved by the AMC—Medical Ethical Committee (Academic Medical Center Amsterdam, The Netherlands; April, 2009; METC 09/046).

**Fig. 1:**
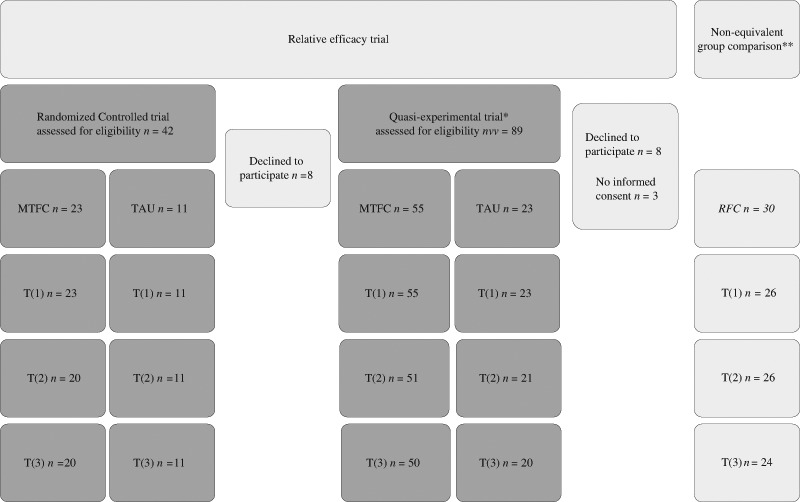
Flow-chart. * Numbers of participants (*n*) presented in this column include all participants that participated in the Randomized Controlled trial. **Participants presented in this column are all regular foster care children. For the non-equivalent group comparison these participants are compared to MTFC-P participants listed under the column Quasi-experimental trial

As implementation of the original trial protocol commenced, clinical objections to the original procedure and financial pressures for the child mental health provider necessitated a change in strategy. The planned ratio (1:1) did not fit the actual treatment capacity as a second team of workers trained in MTFC-P became available. Because ethical considerations eliminated the possibility of a waiting list, the ratio of random assignment to the two conditions was changed to 2:1. The ratio was determined based on the expected number of admissions to the department per year (*n* = 33). However, the actual number turned out lower (*n* = 19). Unused MTFC-P capacity would involve financial risks for the provider, forcing an end to random assignment. As a result, the research design for a large proportion of the sample (see Fig. 1) was no longer compliant with the principles of a randomized controlled trial (RCT), but rather with a quasi-experiment. Treatment outcomes are therefore considered according to two trial designs, as randomized trial and as quasi-experimental trial.

### Method Participants

Children between 3 and 7 years, indicated for permanent foster care placement were eligible to participate. Contrary to short term foster care, children in permanent foster care are expected to stay in the same foster family until adulthood. Children were recruited between June 2009 and January 2013 from the therapeutic foster care service of De Bascule (Amsterdam, The Netherlands). Children were recruited via child protective services and foster parents. Because of their age children were not asked for informed consent. Persons with parental authority over the child as well as foster parents received information when their child was assigned to the service. After approximately one week, they were contacted by researchers to make sure all was understood and asked them to sign informed consent. Whether randomized or non-randomized, eligible children were considered to be study of the quasi-experimental study. The RCT, on the other hand, comprised children that were randomized exclusively. There was no financial incentive for participation.

### Procedure

#### Sample size

Power analysis was based on prevalence of disturbances of attachment in a TFC pilot sample (see Jonkman et al. 2013) and conducted with 90 % power, using Fisher’s exact test with a .05 two-sided significance level. The estimated sample size was 34, but we expected a 10 % cross-over rate and therefore planned to include 40 children per condition.

#### Blinding

Group allocation was disclosed to participants, social workers, and members of the treatment team immediately after referral. Researchers responsible for coding remained blind to group allocation.

### Interventions

#### Multidimensional Treatment Foster Care for Preschoolers

Treatment consisted of an intensive 12 h pre-training for therapeutic foster parents (phase 1), after which children were placed in the therapeutic foster family for nine months. Phase 2 started when children arrived in the therapeutic foster home. Children were paired with a MTFC-P social skills trainer who met with the child once a week. Meetings took place in the therapeutic foster home, at school, or at the playground and were aimed to train social skills and improve behavioral functioning (duration of meetings depended upon the severity of problems). In addition, on a weekly basis all children within one MTFC-P-team (max. 10) joined therapeutic playgroup sessions to further improve social functioning. The therapeutic playgroup was located at the institution, took 2 h each time and was led by four to five social skill trainers. Concurrently with the playgroup, foster parents joined 2 h sessions led by a foster parent consultant. Sessions were aimed to support and supervise therapeutic foster parents. In addition to these sessions, therapeutic foster parents received daily support by telephone and 24 h on-call staff availability. Phase 2 ended after nine months, when children were transferred from the therapeutic foster home to the permanent foster family. In phase 3, social skill trainers and children maintained weekly contact over a period of three months, to facilitate consistency across the transition and to preserve acquired skills. On a less frequent basis permanent foster parents were introduced into effective parental strategies by a family therapist, until approximately three months later when the treatment was completed.

#### Treatment Foster Care as Usual

The treatment foster care program as usual [in Dutch: “Therapeutische Gezinsverpleging” (Van der Most et al. 2001)] consisted of two phases, one diagnostic phase (1) and one treatment phase (2). Phase 1 started immediately after referral with diagnostic screening of children and foster parents in order to identify risk factors for placement breakdown. Therefore, foster parents received home visits and children were seen by a psychologist or psychiatrist. The subsequent treatment phase (phase 2) was adapted to the diagnosed needs of individual children and their foster families. The general structure of contacts was equal for all foster parents; during two-weekly home visits social workers coached foster parents in order to enhance parental skills. Concurrently, the social worker provided children with individual support. When treatment required more specialized help, the social worker was authorized to arrange specific interventions (for example, trauma therapy or video feedback training). Interventions were provided in addition to the regular contacts by the social worker or specialized therapists. If social contextual risks emerged, for example conflicts between biological and foster parents or problems within the biological family, the treatment model also facilitated systemic family sessions led by a systemic therapist and the social worker.

### Measures

#### Primary Outcomes

##### Behavioral Problems

Severity of behavioral problems was determined based on the report of foster parents and teachers. We administered the Child Behavioral Checklist (CBCL) to foster parents and the Teacher Report Form (TRF) to teachers by postal mail (Achenbach 1991; Achenbach and Rescorla 2000). Internal consistency for the broad-band scales internalizing, externalizing, and total problems of both age versions of the CBCL and TRF ranged from .79 to .97, with exception of CBCL 6–18 total scale at the fourth measurement. Internal consistency could not be determined for this scale because insufficient cases were available. Psychometric properties of Dutch versions of the questionnaires have been found acceptable to good (Koot et al. 1997). To merge the different age versions of the CBCL and the TRF t-scores were calculated for the internalizing, externalizing, and total scale.

The Parent Daily Report (PDR; Chamberlain et al. 2006) is a telephone interview with foster parents that was conducted 3, 6, and 9 months after start on five consecutive weekdays. The interview was used to assess the occurrence (0 = *not occurred*, 1 = *occurred at least once*) of 38 problem behaviors within the past 24 h. The average number of daily occurrences per time point was calculated as score = ∑_*n*=5_^PDR^/5 to construct the total problem scale. The PDR has previously been used as a measure for treatment outcomes and psychometric properties have been found adequate (Chamberlain et al. 2006).

### Secondary Outcomes

#### Disturbances of Attachment

To determine if children showed symptoms of disturbed attachment the Disturbances of Attachment Interview (DAI; Smyke and Zeanah 1999) was administered by telephoning with foster parents 3 and 9 months after start of treatment, by trained interviewers. The DAI is a semi-structured interview that consists of 12 items. Five items to check if symptoms of inhibited attachment were present, three items to check if symptoms of disinhibited attachment were present and four items to check for secure base distortions. This study adhered to a dichotomous scoring (0 = *symptom not or somewhat present*, 1 = *symptoms definitely present*), to identify children with clinical symptoms on the scales inhibited attachment, disinhibited attachment, attachment disorder (classifications of inhibited and disinhibited aggregated), and secure base distortions. Item 4 has been found to insufficiently load on any of the DAI subscales (Oosterman and Schuengel 2007a), therefore it was excluded from this study. Previous research has revealed acceptable validity and internal consistency (Smyke et al. 2002; Zeanah et al. 2004).

#### Trauma Symptoms

A Dutch translation of the Trauma Symptom Checklist for Young Children, the TSCYC (Briere et al. 2001; Lamers-Winkelman 1998) was used to screen for trauma symptoms. Based on this questionnaire, foster parents reported how often 90 experiences happened to their child within the last month (1 = *not at all* to 4 = *very often*). The composite score of all items was used to compute a PTSS-total scale (internal consistency *α* = .74–.88). Questionnaires were sent to the foster parents’ home every 3 months, from start to end of treatment. In previous studies the TSCYC demonstrated good reliability (Briere et al. 2001), and moderate convergent and discriminant validity (Lanktree et al. 2008). *T*-scores were calculated, using Dutch norm data (Tierolf and Lamers-Winkelman 2014).

#### Caregiving Stress

The extent of caregiver stress was determined based on a shortened and translated version of the Parenting Stress Index (Abidin 1997), the Nijmeegse Ouderlijke Stress Index-kort (Brock et al. 1992), which consisted of 25 items, rated on a 6-point scale (1 = *totally disagree* to 6 = *totally agree*). Questionnaires were sent to the foster parents’ home every three months, from start to end of treatment. Internal consistency of the questionnaire was .95 at all time-points.

#### HPA-Axis Functioning

Salivary samples were collected 3, 6, and 9 months after start on five consecutive weekdays. Foster parents collected samples immediately after wake-up, 30 min after, and before going to bed. The samples were obtained with a cotton collection device (Salivette; Sarstedt, Rommelsdorf, Germany). On the sampling days, foster parents filled out a brief questionnaire regarding sampling times, stressful events, eating, and sleeping behavior. Analyses were performed by the Cortisol lab in Trier (University of Trier), using a competitive solid phase time-resolved fluorescence immunoassay with flouromeric endpoint detection (DELFIA). To correct for the amount of cortisol that was retained by the cotton, 20 unused reliability salivates were analyzed with a definite cortisol concentration (Hansen et al. 2008).

### Data Analyses

Analyses were performed using the software package SPSS (Statistical Package for the Social Sciences), version 21.0. First, to determine whether changes in behavioral functioning in course of the intervention were different per treatment, we utilized multiple regression analyses. To test for interaction effects, series of repeated measures ANOVA were performed. The remainder of continuous outcomes, measured at >2 time-points were entered into linear Mixed Models. Changes in proportions of symptoms of attachment disorder in course of the treatment and differences between the treatments were examined with Pearson’s Chi-square tests and Fisher’s exact test. Per protocol analyses were performed based on data from the quasi-experimental study sample. Analyses were then repeated for data that derived the RCT sample. Instead of using per protocol analyses, data from the RCT sample was analyzed according to the principles of intention-to-treat, whereby we carried the last observed values forward. This method was used to replace missing values, in order to account for treatment drop-out in the multiple regression analyses. For the actual analyses, this implied that for two participants CBCL and TRF data from the first measurement were also used for the last measurement.

## Results

### Participant Flow

Between mid-2009 and begin-2013, 99 eligible children were assigned to therapeutic foster care (see Fig. 1). The quasi-experimental sample comprised 78 children, 55 MTFC-P and 23 TAU and data were analyzed per-protocol (see Table 1). From this number, 34 children could be included in the randomized controlled trial (RCT). Following intention-to-treat, 23 children were analyzed within the MTFC-P group, 11 children in TAU.

**Table 1:** Baseline demographics

		Total (*n* = 78)	MTFC-P (*n* = 55)	TAU (*n* = 23)	*p*
Sex child (=male)	*n* (%)	50 (64)	35 (64)	15 (65)	.89
Age child (months)	*M*(SD)	63.51 (12.11)	63.36 (12.79)	63.87 (10.58)	.87
Age (months) at out of home placement	*M*(SD)	36.26 (20.72)	38.45 (20.37)	31.00 (21.04)	.15
Time in current family (months)	*M*(SD)	6.54 (13.57)	2.38 (8.56)	16.48 (17.83)	.00
Placement failure	*M*(SD)	4.15 (1.96)	4.22 (1.92)	4.00 (2.09)	.66
Physical abuse	*n* (%)	22 (28)	20 (36)	7 (30)	.62
Neglect	*n* (%)	58 (74)	47 (85)	18 (78)	.44

*Note* *p* = probability of differences between MTFC-P and TAU according to *χ* ^2^ or independent samples *t*-tests

### Preliminary Analyses

At baseline no group differences were found with respect to age, gender, early adverse experiences, and behavioral functioning (see Table 1 and 2). Groups did differ for the duration of stay in the current foster family at the start of the treatment. Children in MTFC-P had been placed in the foster family more recently. Longer time in current family was positively associated with outcomes on the PDR (*r* = .37, *p* < .01) and negatively associated with the teachers’ report of externalizing problems (*r* = −.31, *p* < .05). Time in current foster family was therefore treated as a covariate. Slightly more boys than girls participated in the study, yet the ratio was equal for both groups.

**Table 2:** Descriptive and test statistics for behavioral problems at baseline

		Treatment as usual			MTFC-P			RFC						
	*T*	*N*	*M*	SD	*n*	*M*	SD	*n*	*M*	SD	*t* ^1^	*p* ^1^	*t* ^2^	*p* ^2^
Foster carer report of behavioral problems														
Internalizing	0	23	58.80	16.86	54	59.90	14.80	30	49.37	8.05	−.29	.78	4.23	.00
	1	19	53.28	12.70	46	56.04	11.95	25	50.64	11.09	−.83	.41	1.86	.07
Externalizing	0	23	63.70	20.91	54	60.65	18.85	30	51.80	12.32	.64	.53	2.62	.01
	1	19	53.28	12.70	46	63.22	15.88	25	50.09	9.98	−.20	.84	4.27	.00
Total	0	23	62.72	19.47	54	61.06	15.92	30	50.67	10.75	.39	.70	3.55	.00
	1	19	60.32	15.49	46	61.43	13.94	25	50.17	10.51	−.28	.78	3.53	.00
Teacher report of behavioral problems														
Internalizing	0	15	50.80	7.51	39	50.99	9.97	22	44.94	3.93	−.07	.95	3.36	.00
	1	15	51.53	7.94	35	49.66	10.10	19	45.89	7.66	.64	.53	1.42	.16
Externalizing	0	15	59.04	12.33	39	61.95	15.76	22	52.51	13.79	−.64	.52	2.35	.02
	1	15	59.11	11.80	35	63.57	14.28	19	51.02	9.43	−1.06	.29	3.44	.00
Total	0	15	56.51	10.08	39	58.99	13.59	22	49.52	8.76	−.64	.52	3.30	.00
	1	15	57.79	9.21	35	59.94	12.18	19	48.56	7.90	−.61	.54	4.15	.00

*Note* *T* = time (0 = pre-treatment, 1 = post-treatment). *t* ^1^ and *p* ^1^ = *t*-values and probability of differences between MTFC-P and TAU according to independent samples *t*-tests. *t* ^2^ and *p* ^2^ = *t*-values and probability of differences between MTFC-P and RFC according to independent samples *t*-tests

### Per Protocol Analyses

As illustrated in Table 2, at baseline no significant differences were found in behavioral problems as reported by foster parents and teachers. In addition, no differences were found between MTFC-P and TAU post treatment. A time effect was found with regard to foster parents’ report of internalizing problems, *F*(1, 62) = 6.09, *p* = .02 (see Table 3). However, absence of interaction effects (*p* > .32) suggests that behavioral changes in course of the intervention were similar for children in both conditions. Additional covariate analyses correcting for time in current foster family revealed similar outcomes, *∆R* ^2^ ranged from .00–02, *p* > .23.

**Table 3:** Regression model predicting behavioral problems posttest

	Internalizing					Externalizing					Total				
	*B*	SE *B*	*β*	*∆R* ^2^	*p*	*B*	SE *B*	*β*	*∆R* ^2^	*p*	*B*	SE *B*	*β*	*∆R* ^2^	*p*
Foster carer report of behavioral problems															
Step 1	.25	.09	.32	.11	.01	.56	.10	.58	.32	.00	.44	.10	.51	.26	.00
Step 2	2.30	3.13	.09	.01	.47	2.79	3.84	.08	.01	.47	1.64	3.42	.05	.00	.63
Teacher report of behavioral problems															
Step 1	.69	.16	.60	.36	.00	.57	.12	.62	.39	.00	.59	.14	.58	.35	.00
Step 2	−.87	2.80	−.04	.00	.76	4.58	3.74	.16	.03	.23	2.20	3.36	.09	.01	.52

*Note* Step 1 = pretest score; Step 2 = group (treatment as usual is coded 0, MTFC-P is coded 1)

Results demonstrated that changes over time in trauma symptoms differed between the two treatments (see Table 4). A significantly steeper increase in severity of trauma symptoms from time point one to three was reported in TAU, compared to MTFC-P (*p* = .03). While scores in MTFC-P remained stable, trauma symptoms in TAU showed improvement from time point three to four (*p* = .010). Split-file analyses revealed a time-effect on trauma symptoms changed significantly (*p* = .00), only in TAU. Results demonstrated a group effect on PDR scores, children in MTFC-P showed significantly less problem behaviors on all time-points. A trend towards a significant time effect was also shown, *F*(2, 63.43) = 3.13, *p* = .05. Results revealed significant less problem behavior over time in MTFC-P *F*(2, 49.41) = 4.40, *p* = .02), not in TAU when analyses were repeated within the two groups separately. No differences in effectiveness on diurnal cortisol activity from either children or foster parents were reported. No post treatment differences were found (*p* > .18) with regard to proportions of symptoms of inhibited attachment (*χ* ^2^ = 1.82), symptoms of disinhibited attachment (*χ* ^2^ = .12), symptoms attachment disorder (*χ* ^2^ = .59), and secure base distortions (*χ* ^2^ = .35).

**Table 4:** Descriptive and test statistics per protocol analyses

		*T* (1)			*T* (2)			*T* (3)			*T* (4)				
	tx	*n*	*M*	SD	*n*	*M*	SD	*n*	*M*	SD	*n*	*M*	SD	*F*	*p*Ttx
NOSI-k	1	35	31.62	27.01	36	32.67	22.51	29	32.20	22.85	31	37.80	27.70	–	–
	2	18	36.12	29.32	17	33.86	25.81	11	28.64	28.18	13	26.31	24.91	2.18	.09
TSCYC	1	53	58.21	13.11	50	58.54	10.90	44	57.36	10.57	49	57.90	12.81	–	–
	2	22	54.45	10.48	20	60.50	1442	19	62.68	15.42	19	55.63	11.71	3.21	.02
PDR	1	n.a.	n.a.	n.a.	51	6.20	4.80	49	5.04	3.87	52	5.73	4.48	–	–
	2	n.a.	n.a.	n.a.	17	12.02	9.66	14	13.23	12.26	13	10.35	10.74	1.38	.26
Child-cortisol^a^	1	n.a.	n.a.	n.a.	16	9.33	3.18	16	11.12	6.69	14	11.90	5.73	–	–
	2	n.a.	n.a.	n.a.	16	7.74	3.15	16	9.32	6.53	14	8.98	4.90	1.27	.29
Carer-cortisol	1	n.a.	n.a.	n.a.	16	12.49	5.18	16	11.44	5.80	14	9.67	2.24	—	—
	2	n.a.	n.a.	n.a.	16	10.08	5.98	15	8.39	6.29	14	7.59	3.45	1.20	.32

*Note* n.a. = not available; tx = group (1 = MTFC-P, 2 = TAU); Ttx = treatment effect. *p* = probability of differences between MTFC-P and RFC according to linear mixed models; *p*Ttx = probability of a treatment effect

^a^ nmol/L

### Intention to Treat Analyses

Children in the MTFC-P and TAU did not significantly differ from each other at baseline, with regard to gender (*p* = *.*84), age (*p* = .98), and dependent variables (*p* = .10–.83). Unlike within the per protocol analyses no treatment effect was found with regard to trauma symptoms, *F*(3, 78.40) = .23, *p* = .87. However, a time-effect was found on trauma symptoms, *F*(3, 78.40) = 3.75, *p* = .01. Split-file analyses revealed a significant positive effect of time on decreasing trauma symptoms in TAU, *F*(3, 27.46) = 3.54, *p* = .03, not in MTFC-P. On all other domains no significant other time, group or interaction effects were found using intention to treat analyses, nor when repeated according to the principles of Last Observation Carried Forward.

## Study 2

In addition to the registered RCT, a non-equivalent group comparison design with repeated measures was planned to compare the outcomes between children who received MTFC-P and children in RFC. The treatment group consisted of children from study Study I, treated with MTFC-P. Based on the expected number of MTFC-P participants and an 1:1 inclusion ratio, we planned to include 40 children from regular foster care. The assignment to MTFC-P was according to the strategies described for Study I, the assignment to RFC was not directed by research strategies (observational).

### Method Participants

Children between three and seven years indicated for permanent foster care placement were eligible to participate. Children were recruited between April 2011 and January 2013 from a regular foster care agency, Spirit! (Amsterdam, The Netherlands).

### Procedure

After assignment to the regular foster care agency foster parents received information about the study. After approximately one week, researchers contacted foster parents to make sure all was understood and if they were willing to sign informed consent. There was no financial incentive for participation.

#### Intervention

Regular foster care involved low-frequent contact between foster parents and foster care workers that was only intensified when children were transferred from or to a foster family. Contacts were foster parent oriented, children received little or no intervention.

### Measures

Within regular foster care, behavioral functioning was assessed using the CBCL and TRF as described for Study I. Trauma symptoms were determined with the TSCYC and the NOSI-k was used to inventories the degree of caregiving stress. Questionnaires were sent to the foster home or school of the children every three months from the moment they entered the study, until nine months later. Three and nine months after participants entered the study, we interviewed foster parents by telephone, to determine of children showed symptoms of disturbed attachment (DAI).

### Data Analyses

Study II utilized similar techniques to analyze data, but then changed the independent grouping variable in order to compare children in MTFC-P (*n* = 55) with children in regular foster care (*n* = 30). First, to determine whether there were significant treatment effects on CBCL and TRF scales, accounting for baseline differences and potential regression to the mean, multiple regression was performed. With regard to the other continuous outcomes, measured at four time-points linear Mixed Models was used. Series of *χ* ^2^ tests and independent samples *t*-tests were performed to examine if children after completing MTFC-P leveled with children in regular foster care.

## Results

### Participant Flow

All children within the quasi-experimental sample assigned to MTFC-P were included in the study (*n* = 55) and compared to all 30 children assigned to regular foster care (see Table 1).

### Preliminary Analyses

No significant differences were found between children in MTFC-P and RFC with regard to age at start (*p* = .67) and gender (*p* = .26). At baseline, internalizing, externalizing, and total problems were significantly more severe in MTFC-P, compared to RFC (see Table 2). In addition, baseline differences were significant regarding caregiving stress, *t*(55.62) = 2.10, *p* = .04) and showed a nonsignificant trend regarding trauma symptoms, *t*(81) = 1.83, *p* = .07. We found no significant baseline differences (*p* = .06 – .60) for proportions of children with symptoms of inhibited attachment, disinhibited attachment, and attachment disorder. Fisher exact test was used for secure base distortions and showed significant differences at baseline between MTFC-P and RFC (*χ* ^2^ = 4.42, *p* = .04), with children in MTFC-P showing more secure base distortions.

### Non-Equivalent Group Comparison

Treatment improved the prediction of externalizing problems post treatment as reported by foster parents *R* ^2^ = .37, *∆R* ^2^ = .06, *F* (1, 66) = 5.96, *p* = .02, in MTFC-P problems increased whereas in RFC problems increased. When looking at teachers’ reports of externalizing problems no significant treatment effect was found *R* ^2^ = .46, *∆R* ^2^ = .05, *F* (1, 38) = 3.43, *p* = .07 (see Table 5). Repeated measures ANOVA revealed an interaction effect on the CBCL internalizing scale, *F*(1, 39) = .00, *p* = .03, but no interaction effects on any of the other domains of behavioral problems. Results indicated no treatment effect on trauma symptoms (*p* = .66) or caregiver stress (*p* = .41). However, caregiving stress was significantly higher in the MTFC-P group at baseline, but at the end of the treatment, group differences were no longer significant, *t*(47) = .93, *p* = .37. With regard to attachment, children in MTFC-P showed significantly more secure base distortions at baseline, but post treatment differences were not significant, *χ* ^2^ = .72, *p* = .40. Percentages of children with symptoms of disinhibited attachment and the aggregated indicator of attachment disorder were nearly equal between MTFC-P and RFC at baseline, while post treatment percentages were significantly higher in the MTFC-group, respectively *χ* ^2^ = 4.59, *p* = *.*03 for symptoms of disinhibited attachment and *χ* ^2^ = 6.79, *p* = *.*01 for symptoms of attachment disorder.

**Table 5:** Regression model predicting behavioral problems posttest

	Internalizing					Externalizing					Total				
	*B*	SE *B*	*β*	*∆R* ^2^	*p*	*B*	SE *B*	*β*	*∆R* ^2^	*p*	*B*	SE *B*	*β*	*∆R* ^2^	*p*
Foster carer report of behavioral problems															
Step 1	.46	.09	.55	.29	.00	.48	.10	.48	.31	.00	.45	.10	.49	.31	.00
Step 2	.69	2.73	.03	.00	.80	−8.03	3.29	−.25	.06	.02	−5.12	3.07	−.18	.03	.10
Foster carer report of behavioral problems															
Step 1	.72	.16	.63	.38	.00	.51	.10	.59	.43	.00	.57	.13	.58	.44	.00
Step 2	.61	2.71	.03	.00	.82	−6.60	3.56	−.23	.05	.07	−4.97	3.41	−.19	.03	.15

*Note* Step 1 = pretest score; Step 2 = group (MTFC-P is coded 0, RFC is coded 1)

## Discussion

The purpose of this study was to examine the relative efficacy of MTFC-P compared to an usual treatment foster care program. Practical and ethical considerations led us to deviate from planned research strategies. The results reported are based on a smaller-than-planned sample size within the randomized controlled and quasi-experimental trial. Because of this smaller sample size, true effects had less than desired chances of being detected. Conclusions can therefore only be drawn with caution, indicating that at least MTFC-P did not show very strong benefits over treatment as usual in this current study sample. Comparing MTFC-P and regular foster care, baseline imbalances were inevitable, leaving open the possibility that the reduction of symptom levels in the MTFC-P group to RFC levels may be due to regression to the mean.

Firstly, this study intended to examine changes in behavioral and relationship functioning in course of MTFC-P and compare these with changes in course of the treatment foster care as usual. Results showed that, in the Netherlands, MTFC-P was not superior to treatment foster care as usual in treating behavioral problems, symptoms of attachment disorder, foster parent stress, and neurobiological functioning of children and foster parents. Surprisingly, whereas trauma symptoms in MTFC-P remained almost stable, the first 6 months in treatment suggested negative treatment effectiveness for the usual treatment foster care program. Then the last 3 months, however, showed an advantage of the treatment foster care as usual above of MTFC-P. The substantial size of this latter decrease in trauma symptoms compensated for the previous increase and led to an overall treatment effect in favor of the usual treatment foster care.

Secondly, this study intended to evaluate MTFC-P treatment goals including reduction of symptoms that allow end of treatment and placement in regular foster care. Changes in behavior, trauma symptoms, symptoms of attachment disorder, and caregiving stress were compared between children in MTFC-P and children in regular foster care. Whereas problems were significantly more severe at baseline in MTFC-P compared to RFC, post treatment differences were no longer significant with regard to caregiving stress and secure base distortions. However, percentages of symptoms of disinhibited attachment and attachment disorder were nearly equal between MTFC-P and RFC at baseline, while post treatment percentages became significantly higher in MTFC-P. With respect to externalizing problems as reported by foster parents, children in regular foster care showed a decrease in problems from pre to post treatment whereas children in MTFC-P showed an increase in externalizing problems from pre to post treatment. Significant pretreatment differences between groups on externalizing problems not only continued to exist post treatment, but also increased from pre to post treatment assessment (see Table 2).

In sum, our findings failed to demonstrate relative efficacy for MTFC-P above existing treatment foster care. Children receiving MTFC-P also did not improve in their behavioral problems and symptoms of attachment disorder to the level of problems that can be found among children placed in regular foster care. These findings were unexpected based on positive effects reported in previous studies (Fisher et al. 2005, 2007; Fisher and Kim 2007; Fisher and Stoolmiller 2008). MTFC-P may indeed be effective compared to TAU used in the US, but not compared to the TAU used in the Netherlands. The comparison treatment in the Dutch study comprised usual treatment foster care services, which are tailored to the needs and possibilities of children and foster parents referred to treatment from RFC. Based on the exposure to diverse and complex childhood adversities (see Table 1) need for trauma and/or attachment therapy in addition to behavioral therapy can be expected. Positive outcomes on behavior and trauma suggest that these usual treatment foster care services may include some important mechanisms for effectiveness. Within the eclectic character of usual treatment foster care, social workers can draw on a number of evidence-based interventions and may be able to meet some of the individual needs of children in foster care. The usual treatment foster care program used in this study comprised several evidence-based treatment modules and intensive support for foster parents such as Trauma Focused-Cognitive Behavioral Treatment (TF-CBT; Cohen and Mannarino 2008), Parent Child Interaction Therapy (PCIT; Eyberg et al. 1995), and Eye Movement Desensitization and Reprocessing (EMDR; Shapiro and Maxfield 2002). The comparison services in the US may have comprised less evidence-based and intensive treatment strategies, which enables MTFC-P to show greater effect sizes.

Contrary to the eclectic approach of the usual treatment foster care services in the Netherlands, the strictly protocol-led MTFC-P method may hamper therapists to sufficiently meet the heterogeneous needs of the foster children in the Dutch treatment foster care system. MTFC-P was served to different populations studied in the two trials. Although maltreatment rates and days in care were about similar in the two populations, children in the Dutch population were on average older and had experienced about one more placement failure, compared to the US population (P. Fisher, personal communication, December 2, 2014). Prolonged exposure to insecure caregiving and higher instability may have led to more severe symptomatology in the Dutch population. Unfortunately, traumatic impact of previous experiences has not been determined in ways of symptoms of attachment disorder and PTSD symptoms in US studies and it remains unclear whether MTFC-P in the Netherlands served children with more severe problems.

Besides differences at population-level, the strict protocols of evidence based interventions like MTFC-P, may not be flexible enough to overcomes differences at the level of agencies and countries. MTFC-P is based on extensive research towards the needs of young children in foster care in the United States and experiences with the US foster care system. The inability of MTFC-P to outperform the treatment as usual in the Netherlands may be the result of contextual factors wherein the current trial was performed, which are different from the US. A first major difference is the permanency planning for children in foster care, whereby children in the Dutch foster care system may experience longer-term absence of a perspective on permanency than children do in the US foster care system. In the US, children are regularly adopted, whereas in the Netherlands adoption of children in foster care is rare. Unlike in the Netherlands, in most states of the US parental rights are terminated after children have spent 15 out of the 22 previous months in foster care (Child Welfare Information Gateway 2013). Previous research showed that none of the permanent adoptive family placements of children in MTFC-P failed within 24 months (Fisher et al. 2005). Placement failed in 10 % of the cases which concerned reunification with birth families. Although not investigated in the current study, adoption may bring along more certainty about the future perspectives of children and permanent foster parents, and increases the benefits of the aftercare provided within the MTFC-P intervention. It has been suggested that a lack of permanency planning negatively affects treatment outcomes and therefore the development of children in foster care (Weterings 2000).

### Limitations

A first limitation refers to the smaller-than-planned sample size, limiting statistical power to find differences between active treatments. A second limitation is that treatment compliance was not examined, leaving open the possibility that lack of compliance may have attenuated the advantages of MTFC-P. However, to ensure treatment adherence, complete implementation services were provided for the Dutch MTFC-P staff by TFC Consultants, Inc. (see http://mtfc.com). TFC Consultants, Inc. has set standards that the Dutch MTFC-P staff had to achieve, before this team was certified and allowed to use the name Multidimensional Treatment Foster Care. A third limitation is the absence of a control group without active treatment. The comparison between MTFC-P and regular foster care was hampered by the significant differences at baseline. We used regression methods that accounted for baseline scores, yet it is possible that factors associated with referral are predictive for treatment outcomes. Children in MTFC-P have been referred because of the complexity of their problems, which may have limited their possibilities for improvement. A fourth limitation is the absence of a follow-up measurement to examine long-term effects.

To conclude, the results obtained within this study indicate that children in MTFC-P and usual treatment foster care gained similar outcomes in the Dutch context and this study was unable to replicate positive outcomes of MTFC-P effectiveness in the US. Considering the specific context of the study and the strengths and limitations, the question remains if behavioral improvement is sufficient to change the adverse course on numerous other domains in children’s development. Also, the time effect on behavior in both interventions indicates that not only strict behavioral approaches will lead to behavioral improvement. Extensive research on the effective mechanisms for symptom improvement beyond behavior modification and breaking coercive cycles is therefore needed. Furthermore, based on the findings presented here, still little is known about sustainability of effects on behavior and whether these effects predict foster family placement stability on the long term. Of specific interest is the effect on sustainability of behavioral improvement in MTFC-P, as the end of treatment dictates that children leave the therapeutic foster home. This study should stimulate further development of efforts to restrain the considerable risks of children in foster care with problematic behavior, threatening their placement stability (Oosterman et al. 2007b).
